# Predicting Therapeutic Response to Unfractionated Heparin Therapy: Machine Learning Approach

**DOI:** 10.2196/34533

**Published:** 2022-09-19

**Authors:** Ahmad Abdel-Hafez, Ian A Scott, Nazanin Falconer, Stephen Canaris, Oscar Bonilla, Sven Marxen, Aaron Van Garderen, Michael Barras

**Affiliations:** 1 Clinical Informatics, Metro South Health, Queensland Health Brisbane Australia; 2 School of Public Health & Social Work, Queensland University of Technology Brisbane Australia; 3 Department of Internal Medicine and Clinical Epidemiology Princess Alexandra Hospital Brisbane Australia; 4 Greater Brisbane School of Clinical Medicine University of Queensland Brisbane Australia; 5 Department of Pharmacy, Princess Alexandra Hospital Brisbane Australia; 6 Centre for Health Services Research, Faculty of Medicine, The University of Queensland Brisbane Australia; 7 Pharmacy Service, Logan and Beaudesert Hospitals Logan Australia; 8 School of Pharmacy, University of Queensland Brisbane Australia

**Keywords:** heparin, activated partial thromboplastin time, aPTT, predictive modeling, machine learning, personalized medicine

## Abstract

**Background:**

Unfractionated heparin (UFH) is an anticoagulant drug that is considered a high-risk medication because an excessive dose can cause bleeding, whereas an insufficient dose can lead to a recurrent embolic event. Therapeutic response to the initiation of intravenous UFH is monitored using activated partial thromboplastin time (aPTT) as a measure of blood clotting time. Clinicians iteratively adjust the dose of UFH toward a target, indication-defined therapeutic aPTT range using nomograms, but this process can be imprecise and can take ≥36 hours to achieve the target range. Thus, a more efficient approach is required.

**Objective:**

In this study, we aimed to develop and validate a machine learning (ML) algorithm to predict aPTT within 12 hours after a specified bolus and maintenance dose of UFH.

**Methods:**

This was a retrospective cohort study of 3019 patient episodes of care from January 2017 to August 2020 using data collected from electronic health records of 5 hospitals in Queensland, Australia. Data from 4 hospitals were used to build and test ensemble models using cross-validation, whereas data from the fifth hospital were used for external validation. We built 2 ML models: a regression model to predict the aPTT value after a UFH bolus dose and a multiclass model to predict the aPTT, classified as subtherapeutic (aPTT <70 seconds), therapeutic (aPTT 70-100 seconds), or supratherapeutic (aPTT >100 seconds). Modeling was performed using Driverless AI (H2O), an automated ML tool, and 17 different experiments were iteratively conducted to optimize model accuracy.

**Results:**

In predicting aPTT, the best performing model was an ensemble with 4x LightGBM models with a root mean square error of 31.35 (SD 1.37). In predicting the aPTT class using a repurposed data set, the best performing ensemble model achieved an accuracy of 0.599 (SD 0.0289) and an area under the receiver operating characteristic curve of 0.735. External validation yielded similar results: root mean square error of 30.52 (SD 1.29) for the aPTT prediction model, and accuracy of 0.568 (SD 0.0315) and area under the receiver operating characteristic curve of 0.724 for the aPTT multiclassification model.

**Conclusions:**

To the best of our knowledge, this is the first ML model applied to intravenous UFH dosing that has been developed and externally validated in a multisite adult general medical and surgical inpatient setting. We present the processes of data collection, preparation, and feature engineering for replication.

## Introduction

### Background

Unfractionated heparin (UFH) is a parenteral anticoagulant used for the prevention and treatment of arterial and venous thromboembolic diseases [1,2]. UFH consists of a heterogeneous mixture of polysaccharides with varying molecular lengths and weights; therefore, direct monitoring of serum drug concentrations to guide optimal dosing is not possible [3,4]. Instead, a surrogate of bleeding time, activated partial thromboplastin time (aPTT), is used to monitor the dose-dependent response [5]. The initial bolus and maintenance doses of UFH are estimated by clinicians using weight-based formulas (units of UFH/kg for bolus and units or UFH kg/hour for maintenance), with the aim of achieving a defined therapeutic aPTT range. Future doses are continually adjusted to maintain this therapeutic range (TR) [6-8], which varies depending on the therapeutic indication [1]. An aPTT value below the TR (subtherapeutic) is linked to reduced efficacy (high probability of recurrence or progression of thromboembolic events), whereas values above the TR (supratherapeutic) are linked to the risk of bleeding [9,10]. For patients with life-threatening thromboembolic events, clinicians aim to rapidly achieve a therapeutic aPTT and maintain a TR for the duration of UFH therapy. In the hospital setting, UFH therapy commences with a bolus (loading) dose followed by a maintenance infusion, and an aPPT is quantified within 12 hours [1,6]. This result provides guidance for further dosing, and clinicians often rely on dosing nomograms (Multimedia Appendix 1).

UFH is an extremely complex and difficult drug to accurately dose. The UFH molecules are distributed freely throughout the body; bind to many physiological sites including clotting factors, endothelial cells, and macrophages [4]; and are eliminated from the body via several physiological pathways. This creates marked variation in its pharmacokinetics and dose response between patients, such that there is no standardized *one-dose-fits-all* strategy [8-11]. Despite the use of nomograms to optimize dosing, it is difficult to achieve and maintain a TR that places patients at risk. For example, excessive dosing may result in up to 5.5% of patients having a bleeding event [12]. Studies evaluating metrics of safety and effectiveness, such as time to TR, time within TR, and percentage of patients within TR, have demonstrated an inability to predict optimal dosing with confidence [13-15]. The time to TR after initiation of UFH can be as long as 60 hours in some studies, and a recent local study of 200 patients showed a median time to TR of 36 hours [16]. In another study, only 29% of the patients had 2 consecutive therapeutic aPTTs [15] throughout the duration of treatment. Even in large clinical trials with strict patient monitoring, the percentage of patients attaining aPTT in TR within 48 hours is less than 50% [17-19]. Clearly, many factors influence bodily responses to UFH, which are independent of body weight and are not accounted for in current dosing strategies [7].

### Related Work

Machine learning (ML) is a subset of artificial intelligence that identifies patterns in large data sets and encodes them into models to predict new data [20,21]. ML has great potential for providing decision support tools in modern health care [20,22-24], which are developed using large volumes of digitized patient data contained within electronic health records (EHRs) [25-27]. To achieve optimal dosing of UFH, ML methods can potentially be used to develop models that make accurate predictions for the target aPTT in response to UFH dosing. However, there have been few studies to date on how to use ML to optimize UFH dosing [28]. A recent systematic review [28] identified 8 studies using ML for UFH. Out of these, 4 studies predicted aPTT [29-32]; 1 study [33] reported out-of-TR surrogates for aPTT, including bleeding and clotting events; and the remaining 3 studies [34-36] evaluated UFH dosing in hemodialysis patients [28]. To date, 5 studies [29,30,32,33,36] have been conducted in the intensive care units (ICUs) of hospitals in the United States using retrospective data and 3 studies in the dialysis setting [34-36].

A variety of modeling approaches were reported. Four studies reported supervised learning methods including random forests, adaptive boosting, extreme gradient boosting, and neural networks [30,32,34,36]. One study used an unsupervised approach to train the model, which was then fine-tuned using a supervised approach [34]. Three studies also used regression analysis [29,30,34], 2 studies used a reinforcement learning approach to develop their models [33,36], and 1 study [31] compared neural networks with nonlinear mixed-effects modeling methods. Studies have reported a wide range of performance metrics including accuracy, precision, recall, area under the receiver operating characteristic curve (AUC), *F*_1_-score (a combination of precision and recall), and coincidence rates. The study by Su et al [32] reported the best model accuracy at 88%. Ghassemi et al [29,30] reported 2 studies on modeling for the prediction of UFH dosing, with 1 study reporting a model developed using multinomial regression to predict subtherapeutic and supratherapeutic aPTT, which had superior performance to ML methods. Their later work explored 4 modeling methods, including reinforcement learning and neural networks, with modest accuracies ranging from 0.56 to 0.6. Overall, the multinomial regression model outperformed the ML methods and was a more appealing model because of its clinical interpretability, which is important in the context of implementation and stakeholder engagement. All studies provided limited reporting of reproducibility, and, except for one study by Smith et al [31], none were validated in a new cohort. Most recently, Li et al [37] reported the development and validation of a multiclass aPTT model and subsequent dose prediction application for use in the ICU setting using a shallow neural network approach. The top 5 features for both data sets included a patient’s baseline aPTT, patient weight, total UFH administered, serum creatinine, and age. The model reported performance metrics similar to those of prior studies, with an *F*_1_-score of 0.887 to 0.925. As with prior studies, the study population was limited to ICU patients and may not be generalizable to other clinical settings. It also does not provide guidance on the exact dose changes that clinicians desire at the point of care.

As evidenced by a recent systematic review [28], no identified models had their impact evaluated within routine clinical practice, and research remains limited and of variable quality. In this body of work, we aimed to develop a model that could be used in hospital general medical and surgical wards, which overcomes the limitations of previous studies with regard to methods, reporting, and external validation.

## Methods

### Data Flow

**Figure 1:**
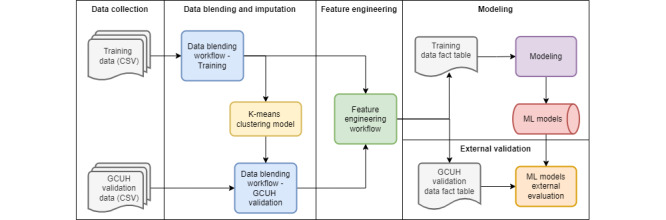
Experiment setup including training and validation processes. GCUH: Gold Coast University Hospital; ML: machine learning; CSV: comma separated values.

Figure 1 depicts the data flow and architecture of the project, which is divided into 5 phases. Phase 1 outlines the data collection in which data files were extracted from EHRs from 5 hospitals, of which 4 were used in model development and the fifth was retained for external validation. In phase 2, the 2 data sets underwent the same phase 2 transformations and mapping process (data blending and imputation), except that the clusters built using the training data set were used unchanged in the validation data set to prevent outcome leakage, where parts of the training data used to create a model were not available at the time of prediction. After data blending, we conducted feature engineering in phase 3, which was again applied to both data sets using the same process. The outputs from the feature-engineering workflow were 2 fact tables: the engineered training data, which were input into the H2O Driverless AI tool to build the ML models (phase 4), and the validation data used to validate the model predictions (phase 5). The same pipeline structure, as shown in Figure 1, was applied in developing and validating a regression model to predict the exact aPTT value and a multiclass model to predict the aPTT class (subtherapeutic, therapeutic, and supratherapeutic), with minor differences in data blending to prepare the outcome columns.

### Ethics Approval

This research work was granted a low-risk research protocol approval and a waiver of consent by the Metro South Health (MSH) Human Research Ethics Committee for ethical and scientific review (reference number LNR/2019/QMS/54581). We confirm that the work completed in this project is consistent with ethics approval of the acquired research.

### Data Collection

EHR data were collected retrospectively for patients admitted between 2017 and 2020 on consecutive admissions to 5 digital hospitals (one health district) in Queensland, Australia, in which UFH was administered for therapeutic purposes. Model development and external validation were undertaken within the Clinical Informatics Division of MSH. We collected data on UFH that were prescribed using a power plan, which is an EHR decision support tool for specific clinical scenarios that facilitates timely ordering of laboratory tests, medication prescribing, and interdisciplinary communication. Four adult-specific power plans that MSH clinicians use, which were used to identify patients eligible for study inclusion, were acute coronary syndrome, deep vein thrombosis or pulmonary embolism, bridging therapy for oral anticoagulants (warfarin replacement), and low–target-range aPTT anticoagulation for neurosurgical patients. This initial patient cohort was then filtered based on the selection criteria defined by the clinician authors:

- Inclusion criteria: adult patients administered a UFH bolus dose and a maintenance infusion for more than 48 hours, had a documented power plan, and had an aPTT result recorded within 12 hours of the UFH bolus dose.
- Exclusion criteria: ICU patients as ICUs use an ICU-specific EHR that is not linked or integrated into the general EHR system in MSH.

A total of 2783 hospital admissions were identified, involving 2470 patients at the 4 hospitals in MSH whose data were used for model development and 236 hospital admissions involving 221 patients at the hospital where data were used for external validation.

Next, we determined the data tables to be collected from the EHRs and generated an initial list of features. Using previous studies in the literature and the content expertise of collaborating clinicians, we identified 15 data tables that were intentionally inclusive at this stage, while recognizing that some would be removed later if found to be irrelevant or if the data were incomplete. Multimedia Appendix 2 lists the tables showing the number of features extracted from each table before and after the feature-engineering phase.

### Data Blending and Imputation

Using the identifier codes of enrolled patients, an aPTT fact table was built by blending UFH bolus dose administration with subsequent aPTT assay results. The rules for inclusion were defined in collaboration between data scientists and clinician researchers to be consistent with the existing literature and to eliminate data noise and ensure data consistency. The following rules were applied:

- A UFH bolus dose was included if it was a de novo (first) dose or was administered after at least 6 hours following prior UFH therapy cessation (equal to approximately 5 UFH elimination half-lives to ensure that no drug remained) [38].
- The aPTT results recorded for the first time after 12 hours of the UFH bolus dose were considered invalid.
- UFH maintenance infusions (maintenance dose) were considered invalid if they were not administered or intravenous infusions were completed, stopped, or paused for more than 1 hour before aPTT testing [39].

**Figure 2:**
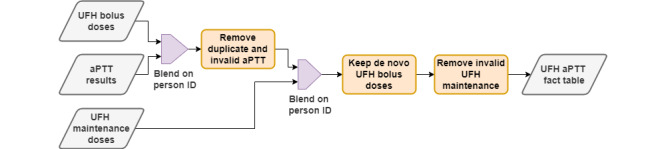
Unfractionated heparin and activated partial thromboplastin time tables blending. aPTT: activated partial thromboplastin time; UFH: unfractionated heparin.

The generation of the aPTT fact table is illustrated in Figure 2. The data blending process was completed in 6 steps, used patient identifiers, and recorded time stamps to connect and filter the data records. The blending process was performed to satisfy the inclusion or exclusion rules previously defined, resulting in a data set of 2158 records for the model development data set and 236 records for the external validation data set.

During the blending of UFH and aPTT data, several features were identified based on clinician input as listed in Textbox 1. For the other tables (Figure S1 in Multimedia Appendix 2), we excluded all records documented after the time the target aPTT had been performed, as derived from the fact table. Looking at the counts, we excluded 3 tables as they had an insufficient number of examples to incorporate into the model, with each having less than (278/2783, 9.98%) of the total records in the fact table. During the blending process, we first identified the columns of interest in each of the remaining tables. For some features, such as age and sex, data were added to the aPTT fact table with minor or no processing. Other features, to be useful, required data to be aggregated, grouped, or converted in some way. For example, a less granular mapping was applied to 166 distinct order catalogs of medications to categorize them into medication classes. However, during modeling, only the antimicrobial group was used because of uneven distributions across the data set for the other groups. A complete feature list with details of the applied processing is included in Table S2 in Multimedia Appendix 2.

Dealing with missing data was the next step after blending all the identified tables into a single fact table (Table 1; Figure S2 in Multimedia Appendix 2 provides more detail on all features and definitions). In general, we used clinician expertise to decide on the imputation methods for achieving the most accurate representation of missing values. Imputing the missing baseline aPTT (Table 1) assumed a normal physiological aPTT value of 30 seconds on the basis of the literature [40] and the median result derived from our training data set. Missing values of patients height and weight were imputed to the mean value of the cohort after grouping by age (bin interval of 10 years), sex, and marital status.

**Features identified during blending of activated partial thromboplastin time (aPTT) and unfractionated heparin (UFH) administration data.**

**Feature and description**

- Baseline aPTT: aPTT result preceding the current (target) aPTT
- Baseline aPTT minutes: time (in) between the baseline aPTT and target aPTT
- UFH bolus minutes: time (in) between the bolus dose and target aPTT
- UFH maintenance minutes: time (in minutes) between the maintenance start and target aPTT

**Table 1:** Missing data handling.

Features	Imputation
Baseline aPTT^a^ and baseline aPTT minutes	Missing values and values completed more than 24 hours before UFH^b^ bolus administration were imputed to 30 seconds, whereas baseline aPTT minutes are imputed to 1440 (24 hours) minutes for those records.
Weight and height	Encounters with no measurements were imputed to the averages for their age, marital status, and sex (as recorded in the patient electronic health record).
Vital Signs features	If the results are missing or occurred more than 12 hours before the target aPTT, they are imputed using centroid values of k-means clustering with k=10.
Pathology results	If the results are missing or occurred more than 1 week before the target aPTT, they are imputed using k-means clustering using centroid value with k=10.
Waterlow score and ADLs^c^	Imputed to 0 where missing or older than 1 week before target aPTT.

^a^aPPT: activated partial thromboplastin time.

^b^UFH: unfractionated heparin.

^c^ADL: activity of daily living.

### Feature Engineering and Data Transformation

In this phase, the blended and imputed aPTT fact table was used initially to conduct univariate analysis and data visualization, which aimed to inform decisions about building new features and transforming data. However, this process was not separate from data modeling; rather, it was an iterative process where ML models were built on initial features that changed and evolved, thus serving as new feature inputs to the next cycle of modeling. Multimedia Appendix 3 provides details and visualizations of Pearson correlations between features and outcomes in our aPTT fact table.

Table 2 summarizes the demographic data and important features that are most relevant to the blended (training) data set. The definitions of diagnoses were based on the International Classification of Diseases (ICD)-10 codes; however, we were only able to include categories with large frequencies; that is, ACS and VTE. Other diagnoses were grouped as other. All patients’ recorded codes during their admission were used in the grouping process.

**Figure 3:**
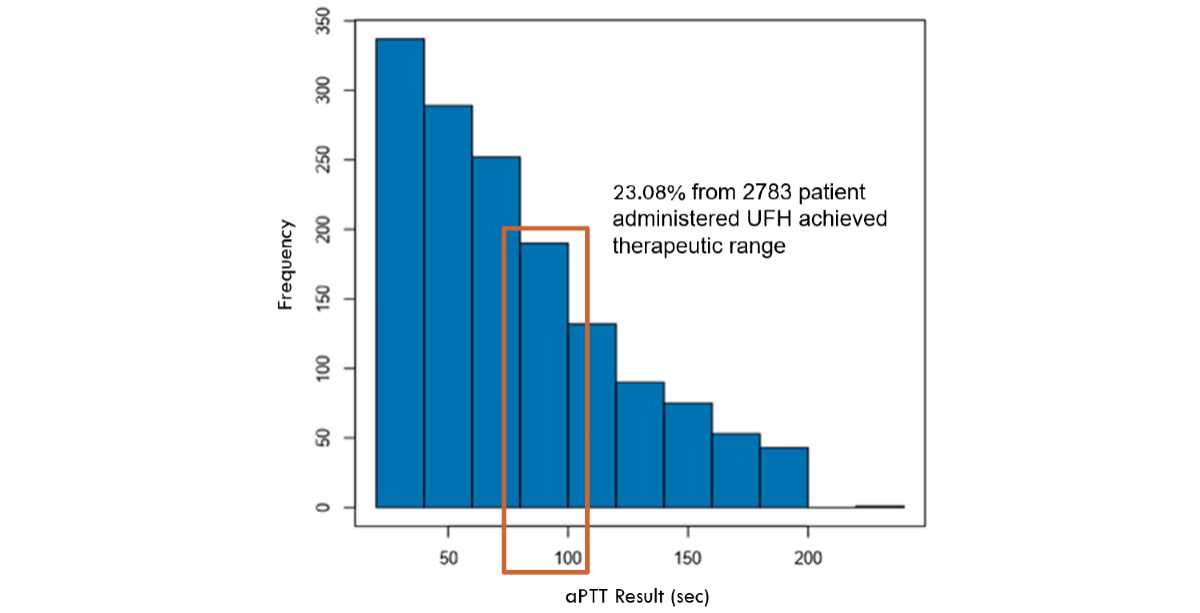
Frequencies of target activated partial thromboplastin time results with bin size=20. aPTT: activated partial thromboplastin time.

The reported aPTT result showed a distribution heavily skewed to the right (Figure 3) and contained outliers, which negatively impacted the performance of a regression model. Although several statistical methods, such as quadratic mean learning [41], can be used to correct this, we chose, on the basis of clinician expertise, to reduce the negative impact of skewness by introducing a floor and ceiling value to target aPTT of 30 and 150. Values less than 30 seconds reflect normal physiological values. The impact of using floor and ceiling values is visualized in residual graphs in Figure S1 in Multimedia Appendix 3, and more feature analysis information is presented in Figures S3-S5 in Multimedia Appendix 2.

Four calculated features were introduced. The first one was the UFH maintenance dose where, unlike the single bolus dose administration, the cumulative maintenance dose was derived based on the total units in the syringe, the infusion period, and the total infusion time before the target aPTT test was performed, excluding any stoppage periods of the infusion (calculation described as follows):


UF*HMaintinance* = (*UFH syringe size / Total infusion period) × (infusion time-infusion stop)*

The standard amount of UFH contained in a syringe was 25,000 units (50 mL syringe, 500 IU/mL), and this, together with the total time for the syringe to be emptied with no interruption, indicated the infusion rate as the number of UFH units infused per minute. The second part of the equation (infusion time—infusion stop) aimed to calculate the exact infusion period in minutes. The 3 other calculated features were body size, UFH bolus time, and UFH bolus time; body size is calculated using the following equations:


Size=*Weight / Height*

UFH Bolus Time = *UFH Bolus Dose / Time to aPTT*

UFH Bolus Time and Size = Size × UFH Bolus Time

Finally, we added a cosine cyclical transformation of aPTT time to build 3 features representing the aPTT day of the week, hour of the day, and month of the year. At the end of this phase, we obtained 93 features in the aPTT fact table. Depending on the data distributions, continuous variables were scaled using the Yeo-Johnson transformation [42] from the SciPy Python library [40] or a min-max transformation. Details about the transformation method applied for every feature are provided in Figure S2 in Multimedia Appendix 2, and equation details are available in Multimedia Appendix 4.

**Table 2:** Baseline characteristics of training data set (all records, N=2783).

Feature			Values
**Sex, n (%)**			
	Male	1898 (68.2)	
	Female	885 (31.8)	
**Diagnosis, n (%)**			
	ACS^a^	818 (29.4)	
	VTE^b^	540 (19.4)	
Age (years), mean (SD)			65.8 (14.6)
Weight (kg), mean (SD)			87.8 (26.7)
Baseline aPTT^c^ (seconds), mean (SD)			36 (11.1)
UFH^d^ bolus dose (units), mean (SD)			4713 (1467)
UFH maintenance (units), mean (SD)			6767 (4993)
Time between UFH bolus and aPTT (minutes), mean (SD)			364.1 (149)

^a^ACS: acute coronary syndrome.

^b^VTE: venous thromboembolism.

^c^aPTT: activated partial thromboplastin time.

^d^UFH: unfractionated heparin.

### Modeling

#### Outcomes and Setup

In this phase, 2 models were developed: a regression model for predicting the target aPTT result and a multiclassification model for predicting the aPTT class as subtherapeutic, therapeutic, or supratherapeutic. To identify the optimal model in each case, several models were iteratively tested, with each iteration evaluated using 3-fold cross-validation involving 67 by 33 data splits, where all cases could be used for both model training and internal validation. Cross-validation was repeated 3 times to ensure that the validation metrics were robust, as the training data sets were relatively small. The predictive metrics of all iterations were averaged to obtain the overall results for the model.

The modeling process was completed using the H2O Driverless AI tool, which is an auto-ML tool that takes tabular data as input and builds supervised models automatically using the available open-source ML libraries in Python and R. It also automates model validation, tuning, and selection to achieve an accuracy level equivalent to that of the manually built models. The tool also performs an iterative feature evolution process during modeling to discover new features. Supervised ML models supported by H2O Driverless AI include XGBoost [43], LightGBM [44], generalized linear models [45], TensorFlow [42], RuleFit [46], and FTRL [47] (followed by the regularized leader) [48]. The tool will generate and test large numbers of models by using different open-source algorithms, undertake hyperparameter tuning, try different feature subsets, and combine models using different methods. After generating hundreds of different models and combinations (ensembles), the tool recommended the most accurate model built for deployment.

#### Ensemble Regression Model

We built an ensemble regression model to predict aPTT values within 12 hours of a UFH bolus dose. The optimized performance metric was the root mean square error (RMSE). Other reported metrics include the mean absolute error and coefficient of determination (*R*^2^).

During this experiment, 1126 alternative models were trained, including constant predictions, the LightGBM [44] and XGBoost [43] algorithms, and ensemble models. After the feature evolution process, the 93 original features were converted into 188 features, with the contributing features on every model automatically selected by the H2O Driverless AI tool during the training process. We built several baseline regression models, against which the performance of the H2O Driverless AI ensemble model was compared. For all these baseline models, we used the same set of features used to build the ensemble model, except for those evolved during the modeling process using the H2O Driverless AI tool. The first baseline model was built using the tool but as a single model rather than an ensemble. The best model returned by the tool is the XGBoost model. The other baseline models were developed using the Python scikit-learn library, where we tested 3 different linear regression models: Ridge [49], Lasso [50], and ElasticNet [51].

#### Ensemble Multiclassification Model

We built a multiclassification model using the same training data set to predict the target aPTT class, where aPTT<70 seconds was considered subtherapeutic, aPTT between 70 and 100 seconds as therapeutic, and aPTT>100 seconds as supratherapeutic. In this modeling process, we optimized the accuracy and reported several other metrics relevant to multiclassification, including macroprecision, macrorecall, macro–*F*_1_-score, and macroaverage one class vs rest classes AUCs [52].

In total, 457 different models were trained and tested using the H2O Driverless AI tool. Similar to the regression models, the tool tested constant predictions, the LightGBM [44] and XGBoost [43] algorithms, and ensemble models. The evolved and original features used in the modeling process totaled 2196 features. The tool ranked the models based on their performance and recommended the best performing model producing the best accuracy. The other baseline models were developed using the Python scikit-learn library: logistic regression, logistic regression with recursive feature elimination, support vector machine [53] using a linear support vector classifier [54], and support vector machine using polynomial support vector classifier.

### External Validation

External validation was performed using data obtained from patient records at a fifth hospital (Gold Coast University Hospital), which had an exact schema and table structure as the training set. The final data set comprised 236 records, after applying the same inclusion or exclusion criteria as the training data set. We used the method proposed by Archer et al [55] to calculate the sample size sufficient to validate the proposed model and achieve a preselected target for the CI of *R*^2^. The equations and calculation details are provided in Multimedia Appendix 4. The equation generated a minimum sample size of at least 235 participants, which was achieved.

## Results

### Ensemble Regression Model

The best performing model was an ensemble of 4x LightGBM models that were linearly blended. LightGBM is a gradient-boosting framework that uses tree-based learning algorithms [44]. The model relied on 134 features, some of which are well-established as influencing responses to UFH, such as weight and baseline aPTT, with others first identified in this experiment, of which the most important was the time between when the bolus dose was administered and when the aPTT was measured (Table 3). The bolus dose time, baseline aPTT, age, and bolus dose were also relatively important. Weight, size (weight divided by height), and hematological and biochemical parameters, including serum creatinine level, as a measure of renal function, were also among the top 20 features. Table 4 shows the description and selected list of the LightGBM hyperparameters.

The performance metrics of our ensemble and all the baseline models are listed in Table 5. The H2O Driverless AI ensemble model had best performance with RMSE 31.35 (SD 1.37), residual charts provided in Figure S1 in Multimedia Appendix 3. In addition, this baseline model outperformed all other Python-based linear regression models because the tool tested several algorithms, as previously mentioned, and evolved additional features during the modeling process.

**Table 3:** Top 10 most important features with relative importance scores.

Feature	Relative importance
Minutes between UFH^a^ bolus and aPTT^b^	1
UFH bolus time	0.58
Baseline aPTT	0.41
Age	0.37
UFH bolus dose	0.3
Height	0.25
UFH maintinance	0.23
UFH bolus size calculated	0.22
Weight	0.21
Size calculated	0.195

^a^UFH: unfractionated heparin.

^b^aPTT: activated partial thromboplastin time.

**Table 4:** Descriptions of contributing models in the final regression ensemble model.

ID	Model type	Model weight	Fitted features	Feature fraction	Max leaves	Learning rate	Max bins	Lambda L1	Lambda L2
0	LightGBM	0.3333	65	0.6	16	0.01	128	0	0.5
1	LightGBM	0.1042	69	0.6	16	0.01	128	0	0.5
2	LightGBM	0.1667	95	0.8	64	0.01	256	0	10
3	LightGBM	0.3958	134	0.4	16	0.01	64	0	2

**Table 5:** Performance of regression models for predicting activated partial thromboplastin time results.

Tool	Model	Mean absolute error	Root mean square error	*R^2a^*
H2O DAI^b^	Final ensemble model	*24.61* ^c^	*31.35*	*0.355*
H2O DAI	XGBoost	25.51	32.33	0.31
SKlearn^d^	Linear regression	26.89	33.8	0.244
SKlearn	Ridge regression	26.93	33.79	0.244
SKlearn	Lasso regression	26.93	33.68	0.249
SKlearn	Elasticnet regression	27.15	33.72	0.247

^a^R^2^ coefficient of determination.

^b^DAI: Driverless AI.

^c^Minimum error rate.

^d^SKlearn: a machine learning library in Python.

### Ensemble Multiclassification Model

The best performing model was a linear blend ensemble of 4 different models with different weights, 2 XGBoost models and 2 LightGBM models (Table 6).

The performance metrics of the ensemble multiclassification model and baseline models built using the SKlearn library in Python are presented in Table 7, with the ensemble model showing superior performance across all metrics, with an accuracy of 0.599 and macro–*F*_1_-score of 0.613. The simple logistic regression model in Python was the second-best performer, highlighting the efficiency of using auto-ML tools for feature engineering, evolution, and model tuning and blending.

**Figure 4:**
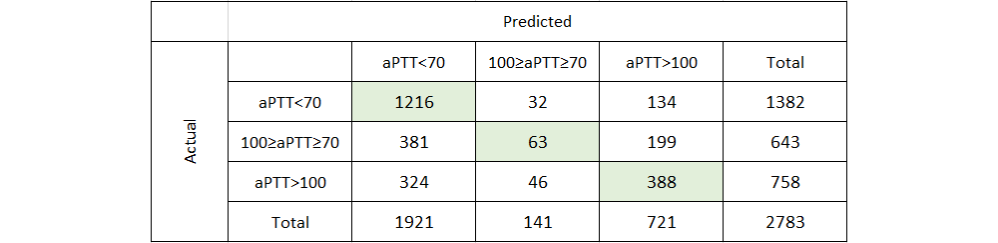
Multiclassification confusion matrix. aPTT: activated partial thromboplastin time.

Figure 4 shows the confusion matrix for the ensemble model, demonstrating very good accuracy (0.88) for the subtherapeutic class aPTT<70 seconds, intermediate accuracy (0.512 for the supratherapeutic class aPTT>100 seconds, and poor accuracy (0.098) for the therapeutic class aPTT 70 to 100 seconds. This lower accuracy is most likely a result of class imbalance due to the underrepresentation of the therapeutic class in the data set. Nevertheless, predicting patients at risk of recurrent thromboembolic events from underdosing or at risk of bleeding from overdosing is important for clinicians and patients.

**Figure 5:**
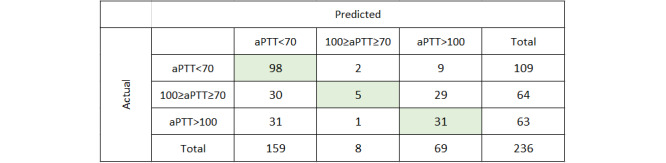
Multiclassification confusion matrix for external validation. aPTT: activated partial thromboplastin time.

For the multiclassification ensemble model, the validation set returned an accuracy of 0.568 (95% CI 0.538-0.598) and an AUC of 0.724 (95% CI 0.714-0.734), which also compares favorably with the corresponding values for the training set cross-validation of 0.599 and 0.735, respectively. In surveying the confusion matrix (Figure 5), the model demonstrated similar accuracy for each class as the training model: 0.899 for the aPTT class <70 seconds, 0.492 for the aPTT class >100 seconds, and 0.078 for the aPTT class 70 to 100 seconds.

**Table 6:** Descriptions of contributing models in the final multiclassification ensemble model.

ID	Model type	Model weight	Fitted features	Feature fraction	Max leaves	Learning rate	Max bins	Lambda L1	Lambda L2
0	XGBoost	0.3067	1900	0.2	8	0.01	128	0	0.5
1	XGBoost	0.2	1914	0.5	8	0.01	256	0	5
2	LightGBM	0.4	78	0.6	16	0.01	64	0	0.5
3	LightGBM	0.0933	183	0.8	64	0.01	256	0	0

**Table 7:** Performance of multiclassification models in predicting activated partial thromboplastin time class.

Tool	Model	Accuracy	Macroprecision	Macrorecall	Macro–*F*_1_-score	Macro-AUC^a^
H2O DAI^b^	Final ensemble model	*0.599* ^c^	*0.554*	*0.686*	*0.613*	*0.735*
SKlearn	Logistic regression	0.562	0.51	0.56	0.52	0.691
SKlearn	Logistic regression with RFE^d^	0.557	0.49	0.56	0.5	0.687
SKlearn	SVM^e^—linear SVC^f^	0.535	0.51	0.54	0.517	0.679
SKlearn	SVM—polynomial SVC	0.451	0.46	0.45	0.457	0.614

^a^AUC: area under the receiver operating characteristic curve.

^b^DAI: Driverless AI.

^c^Best calculated accuracy.

^d^RFE: recursive feature elimination.

^e^SVM: support vector machine.

^f^SVC: support vector classifier.

## Discussion

### Principal Findings

This study reports the development and external validation of an ML model for the prediction of aPTT following bolus and maintenance dosing with UFH. The ML models were developed using EHR data from 4 Australian hospitals with the best performing approach, producing an ensemble with 4x LightGBM models with an RMSE of 31.35. As a multiclassification task, the ensemble model achieved an accuracy of 0.599 and an AUC of 0.735. External validation in a new patient cohort at a fifth hospital showed similar results, with an RMSE of 30.52 for the prediction model and an accuracy of 0.568 and AUC of 0.724 for the multiclassification model.

The final model relied on 93 features, including body weight, baseline aPTT, and bolus dose, with others novel to this study and contemporary clinical knowledge, such as hematological and biochemical features (Multimedia Appendices 2 and 3). The most important clinically informative novel features were the time between administration of the bolus dose and aPTT, age, and baseline aPTT. Baseline aPTT, maintenance UFH dose, and time between bolus administration and aPTT as a grouped feature, which had the highest relative importance (Table 3). UFH is considered a high-risk drug with a narrow therapeutic window, and therefore requires patient-specific dosing to ensure safety and effectiveness [1,7]. Determining the optimal initial bolus and maintenance dosing for UFH therapy is challenging because of the many unknown physiological variables that may contribute to its anticoagulant response. Initial bolus dosing based on body weight is currently preferred [19]; however, other variables must influence the response [56,57]. Nomograms and regular aPTT monitoring, which guide subsequent dose adjustments, increase the proportion of patients achieving a target therapeutic aPTT range [6,8]. Unfortunately, local data derived from 2783 patient episodes suggest that this target is achieved in as few as 23.08% of the patients administered UFH.

As UFH is difficult to administer, new anticoagulants have been introduced in the health setting. Although these new anticoagulants, such as direct-acting oral anticoagulants and low–molecular-weight UFHs, have similar effectiveness to UFH in thromboembolic disease, UFH retains an extensive role in hospital practice because of its several advantages [1,2]. Current dosing is based on nomograms, drug action can be quickly reversed if required, the response can be monitored using aPTT, and its short half-life ensures that the drug is quickly eliminated if urgent surgery is required, or bleeding occurs. As per our data, UFH is still a commonly used drug that requires better dosing and monitoring to ensure patient safety than what is currently achievable. Using ML to derive a predictive model offers a possible approach to more accurately predict individual responses to an initial bolus dose of UFH. This information will assist clinicians in estimating the optimal bolus dose. Developing, testing, and deploying these models is becoming more feasible with the advent of large, digitized data sets such as EHRs [22,26,58]; systems that have been implemented in most large hospitals in Australia. Our study demonstrates that many other features exist beyond the traditional weight-based calculations to determine the best UFH bolus dose. This has the potential to improve the safety profile of UFH. EHR data now afford the opportunity to start using model-based dosing strategies and the ability to develop continuous learning ML models in the future [59].

ML is increasingly being used in early phase drug development [22,26,58] and postmarketing dose design, particularly for other high-risk drugs with narrow therapeutic windows, such as warfarin [56,57,60,61], insulin [62], digoxin [63], immunosuppressants, and chemotherapeutics [64]. Using ML models to guide dosing of UFH in acutely ill, unstable medical and surgical patients to minimize thromboembolic events and bleeding events is an important advancement. In developing such models, as shown in our study, a collaborative approach whereby clinicians and data informaticians work in close consultation is essential. Our study used researchers, data engineers, hematologists, pharmacists, and medical practitioners in its design and conduct. This is essential for developing usable artificial intelligence solutions in hospital settings [65].

### Comparison With Prior Work

In this study, an ensemble approach with supervised learning was used. Five other studies have reported using supervised learning in developing models to assist with UFH dosing [29,30,32,34,36]. To date, although 3 report accuracy [32,33,37] superior to that of our ensemble approach, these models were restricted to ICU data sets from the United States and China and are, therefore, not generalizable to the general medical and surgical wards of hospitals where UFH is most frequently administered. Furthermore, compared with all existing studies of ML in UFH dosing, ours was the only one, apart from one small external validation in a hemodialysis setting [31,66], to evaluate model performance when applied to new unseen data. External validation is considered an essential step before assessing the efficacy in controlled clinical trials and subsequent implementation in routine practice [65,67].

### Future Work

The stage is now set for a feasibility study, the implementation of the model in hospital clinical workflows, and, if successful, further evaluation of clinical utility in a trial comparing current standard practice with model-guided bolus dosing. Implementing the model in routine practice requires an easily accessible decision support platform that can prepopulate most, if not all, the features within the model from the EHR without the need for manual input by clinicians. The model will need to rapidly provide guidance at the exact time of decision-making and will not require end users to undertake extensive training in its use [68,69].

### Limitations

Our model was developed and validated using data from EHRs of 5 hospitals and, therefore, should be tested in other health care systems that use EHRs. The modeling approach only applies to adult inpatients admitted to general medical and surgical specialties. ICU patients were excluded from this study. Furthermore, this modeling approach was intended for the prediction of aPTT after a prespecified bolus and maintenance dose, and as such, further work is required to allow dose calculation and adjustment.

Some features (such as activities of daily living assessments), which were included in the 93 influential features, may not always be available at the time of dosing UFH, and appropriate surrogates should be considered in future iterations of the model. It is also important to consider the level of data standardization within the EHR data sets, which may limit the applicability and usefulness of ML-derived models [70]. For example, differences in how features are measured (eg, the weight and height using different scales), differences in aPTT assays, or different locations of data in EHR may affect model performance and generalizability.

Finally, similar to many other dosing regimens for intravenous drugs, a perfect algorithm for UFH is probably not achievable, as UFH interacts with a myriad of hematological and physiological factors that may affect its anticoagulant effect. Many of these cannot be measured or remain unknown. The goal of our study was to produce and validate a predictive model for UFH dosing that is significantly more accurate than the current weight-based nomograms that have been in use for many years.

### Conclusions

This study reports the development and validation of an auto–ML-built ensemble modeling approach for predicting aPTT results and determining their therapeutic classification within 12 hours of administration of a de novo UFH bolus accompanied by a UFH maintenance infusion. ML models were developed using retrospective data from the EHRs of the 4 hospitals. These models were shown to have a consistent performance when applied to an external data set from a fifth hospital. To our knowledge, this is the first study of ML regression and multiclassification models applied to UFH dosing that has used auto-ML tools in model development and conducted external validation. Future work should include the optimization of model performance and its redesign and incorporation into a dose calculation software tool that can be easily used by clinicians at the point of care.

## Appendix

Multimedia Appendix 1 Dosing and monitoring nomogram used in Queensland, Australia. Retrieved from “State of Queensland (Queensland Health). Heparin intravenous infusion order and administration form for adults. 11th ed. Queensland 2018.”.

Multimedia Appendix 2 Data analysis, description, and processing extra tables.

Multimedia Appendix 3 Data visualizations, heatmaps, plots, and residual graphs.

Multimedia Appendix 4 Metrics equations.

## Abbreviations

aPTT: activated partial thromboplastin time
AUC: area under the receiver operating characteristic curve
EHR: electronic health record
ICU: intensive care unit
ML: machine learning
MSH: Metro South Health
RMSE: root mean square error
TR: therapeutic range
UFH: unfractionated heparin

## Data Availability

Access to the data used in this project is restricted to the research team and for the duration of the research project, as outlined in the national statement on ethical conduct in human research 2007, the Australian code for the responsible conduct of research, and Metro South research management policy and procedures. Access to data was granted by Metro South Health Research Governance Committee, Site Specific Assessment reference number SSA/19/QMS/54581.
