# Supplementary material for: Predicting Therapeutic Response to Unfractionated Heparin Therapy: Machine Learning Approach
Source: Interact J Med Res. 2022 Sep 19;11(2):e34533. doi: 10.2196/34533 (PMC9531006; doi:10.2196/34533)
Supplement: Multimedia Appendix 2 [file ijmr_v11i2e34533_app2.pdf]

Multimedia Appendix 2

Table S1: List of initial data tables collected from electronic health records (EHR) for the participants and whether they were used in blending and modelling.

Database Table	Description	Number of features post blending	Number of features post engineering	Reason for change
Encounters' Data	Patient data such as age, sex	10	11	Medical Service was grouped into three categories (Cardiology, Surgery and General Medicine).<br>Dropped ATSI[c] due to low counts
aPTT[b]	aPTT test times and results	4	6	Cyclical transformation of aPTT time created 3 features.
Pathology	All pathology tests	47	40	Dropped multicollinearity features.
UFH[e]	All UFH administrations	6	8	Added calculated features
Medications	All administered medications	1	1	Medications were grouped with the help of pharmacists to create 3 groups. We dropped antibiotics and sedative due to low counts and kept antimicrobial group.
Locations	Patients ward transfers	2	2	-
ADLS	Activities of daily living data	1	1	-
Diagnosis	Recorded patient diagnoses	1	3	Diagnosis grouped into three categories (ACS[a], VTE[f], and other)
Measurements	Patient weight, height, etc	2	3	Added size (calculated) feature = Weight/ Height
Power-plan	Patient PowerPlan data.	1	4	Powerplan grouped into four categories (ACS, DVT[d], Warfarin, or Low Target)
Vital signs	All patient vital signs	8	8	-
Waterlow	All Waterlow results	6	6	-
Blood Transfusions	All blood transfusions	2	-	Low counts
IV lines	Recorded IV-line insertions	1	-	Low counts
Procedures	All patient procedures	6	-	Low counts
Total		**98**	**93**	

[a]ACS: acute coronary syndrome.

[b]aPTT: activated partial thromboplastin time.

[c]ATSI: Aboriginal or Torres Strait Islander person.

[d]DVT: deep vein thrombosis.
[e]UFH: unfractionated heparin.
[f]VTE: venous thromboembolism.

Table S2: Post engineering features with correlation score to target aPTT

Data source	Feature name	Correlation (*p*-value)	Description (data transformation method used)
Encounters' Data	Age	0.12 (<.01)	Age of the patient (Y-JPT[i])
	Australia/ NZ[f] Indicator	-0.04 (.056)	Whether the patient's country of birth (COB) is Australia or NZ (1) or not (0). If missing it is imputed to 1.
	Cardiology	-0.07 (<.01)	Indicates whether the patient's medical service is cardiology.
	General Medical	0.001 (.95)	Indicates whether the patient's medical service is general medicine.
	Surgery	0.065 (<.01)	Indicates whether the patient's medical service is surgery.
	COB Latitude	0.013 (.53)	Country of birth latitude. If missing imputed to Australia latitude.
	COB Longitude	-0.036 (.09)	Country of birth longitude. If missing imputed to Australia longitude.
	Female	0.089 (<.01)	Indicates whether the patient is female.
	Male	-0.089 (<.01)	Indicates whether the patient is male.
	Hours in Hospital	0.18 (<.01)	Hours since encounter start time. (Y-JPT)
	Married	-0.002 (.91)	Indicates whether the patient is currently married.
aPTT[b]	Day Cosine	-0.02 (.27)	aPTT Date Time converted to a day, hour, and month features using a cyclical transformation (7, 24, and 12)
	Hour Cosine	0.05 (.01)	
	Month Cosine	-0.004 (.84)	
	Baseline aPTT	0.33 (<.01)	The aPTT result preceding the bolus. Imputed to 30 if missing or older than 24 hours.
	Baseline aPTT imputed	-0.24 (<.01)	Indicates whether the baseline aPTT was imputed (1/0)
	Baseline aPTT minutes	-0.24 (<.01)	Minutes between the baseline aPTT and the target. Imputed to 1440 (24 hours) if missing.
UFH[g]	UFH Bolus dose	0.03 (.172)	Bolus dose units (Y-JPT)
	Minutes between UFH bolus and aPTT	0.26 (<.01)	Minutes between the bolus and the aPTT test. (MMT[e])
	UFH bolus time calculated	-0.213 (<.01)	(UFH bolus dose)/ (UFH minutes between bolus aPTT). (Y-JPT)
	UFH bolus size calculated	-0.232 (<.01)	(Measure_Weight / Measure_Height) * (UFH bolus time calculated). (Y-JPT)
	UFH maintenance	0.25 (<.01)	The cumulative maintenance (0-25,000 units) that the patient has received at the time of the aPTT test. Derived from the lapsed proportion of the Infuse_Over time * 25,000. Maintenance stoppage (indicated in the Power-plan table) has been factored into this. (MMT)
	Minutes between UFH	0.24 (<.01)	Minutes between the maintenance start and aPTT test. (MMT)

	maintenance begin & aPTT		
	Minutes between UFH bolus & UFH maintenance begin	-0.099 (<.01)	Minutes between the maintenance start and bolus. (Y-JPT)
	UFH effective bolus	-0.041 (.06)	If UFH maintenance has been stopped for > 90 minutes this value is imputed to 0. If the minutes between maintenance and bolus are between 0 and 90, this value is equal to UFH_Bolus_Dose * (90 – (UFH minutes between bolus & maintenance begin) / 90). (Y-JPT)
Medications	Antimicrobial	-0.003 (.89)	Indicates whether the patient received Antimicrobial or not (1,0)
ADL	ADLs	-0.001 (.94)	Indicates whether daily activities are independent (0) or require assistance (1). Imputed to 0 if missing or older than 1 week.
Locations	Total Locations	0.065 (<.01)	Number of times the patient has changed rooms per encounter. (Y-JPT)
	Unique Locations	0.059 (<.01)	Number of unique locations the patient has been relocated to. (Y-JPT)
Power-plan	ACS[a]	-0.071 (<.01)	Each feature is an indicator of the patient's UFH Power-plan (ACS, DVT, Warfarin or Low Target)
	DVT[d]	0.11 (<.01)	
	Low Target	-0.066 (<.01)	
	Warfarin	-0.018 (.41)	
Diagnoses	ACS	-0.07 (<.01)	Indicates whether the patient has been diagnosed with acute coronary
	VTE[h]	-0.005 (.82)	Indicates whether the patient has been diagnosed with acute VTE
	Other	0.003 (.86)	Other diagnosis
Measurements	Height	-0.081 (<.01)	Patient's measured height. (Y-JPT)
	Weight	-0.11 (<.01)	Patient's measured weight. (Y-JPT)
	Size (Calculated)	-0.086 (<.01)	Measure_Weight / Measure_Height. (Y-JPT)
Vital signs<br><br>(latest results recorded before target aPTT are used. Readings are imputed if missing or older than 12 hours, using centroid values of k-means clustering with k=10)	DBP	-0.038 (.07)	Patient's Diastolic Blood Pressure(mmHg). (Y-JPT)
	SBP	-0.028 (.207)	Patient's Systolic Blood Pressure (mmHg). (Y-JPT)
	Mean AP Cuff Calc	-0.038 (.078)	Patient's Mean Arterial Pressure (MAP) calculated. (Y-JPT)
	Peripheral Pulse Rate	-0.103 (<.01)	Patients pulse rate in beats per minute. (Y-JPT)
	O2 Flow Rate	0.056 (.01)	Flow rate of oxygen (in litres) if patient required . (Y-JPT)
	Assisted O2	-0.007 (.75)	Assisted O2. (Y-JPT)
	SpO2	0.033 (.127)	Blood Oxygen saturation (%).(Y-JPT)
	Temperature	0.011 (.604)	Temperature readings were coalesced into one field to make the data less sparse. Four values of temperature are merged in a priority order as, oral, tympanic, axillary, and rectal. (Y-JPT)
	Albumin Level	0.006 (.78)	Patient Albumin level. (Y-JPT)

Pathology results<br><br>(latest results recorded before target aPTT are used. Results are imputed if missing or older than 7 days, using centroid values of k-means clustering with k=10.)	Alkaline Phosphatase	-0.044 (<.05)	Patient's Alkaline Phosphatase (ALP) level, measured in Units/L. (Y-JPT)
	Anion Gap	-0.028 (.19)	Patient's Anion gap (ie. (sodium+ potassium) - (Chloride + Bicarbonate)) in mmol/L. (Y-JPT)
	Bicarbonate Level	0.021 (.33)	Patient's Bicarbonate level in mmol/L. (Y-JPT)
	Bilirubin Conj	0.053 (.014)	Patient's conjugated bilirubin level (in µmol/L). (Y-JPT)
	C Reactive Protein	-0.014 (.527)	Patient's C-Reactive Protein (CRP) in mg/L. (Y-JPT)
	Calcium Level	0.005 (.79)	Patient's Calcium level in mmol/L (not corrected for albumin). (Y-JPT)
	Chloride Level	0.041 (.06)	Patient's Chloride level in mmol/L. (Y-JPT)
	Cholesterol	-0.034 (.118)	Patient's Total Cholesterol level in mmol/L. (Y-JPT)
	Cholesterol HDL	-0.046 (.037)	Patient's High Density Lipoprotein level in mmol/L. (Y-JPT)
	Creatine Kinase	0.014 (.515)	Patient's Creatinine Kinase (CK) level In U/L. (Y-JPT)
	Creatinine	0.033 (.12)	Patient's serum creatinine level in µmol/L. (Y-JPT)
	cTroponin I	0.029 (.189)	Patient's cardiac troponin I (cTnI) level assay in ng/L. (Y-JPT)
	Eosinophils	0.025 (.23)	Patient's Eosinophil Count, (measured as x 10^9/L cells). (Y-JPT)
	Fibrinogen Derived	-0.037 (.089)	Patient's plasma factor I (fibrinogen) level, derived from Prothrombin time in g/L. (Y-JPT)
	Gamma GT	-0.041 (.06)	Patient's Gamma glutamyltransferase (GGT) level, measured in Units/L. (Y-JPT)
	Globulin	-0.042 (.06)	Patient's globulin (ie. total protein – albumin) level in g/L. (Y-JPT)
	Glucose Level	-0.1 (<.01)	Patient's blood glucose level in mmol/L. (Y-JPT)
	Haemoglobin	-0.04 (.06)	Patient's Haemoglobin (Hb) in g/L. (Y-JPT)
	HbA1c NGSP	0.024 (.268)	Patient's glycohaemoglobin (HbA1c) level (in %). (Y-JPT)
	INR	0.068 (<.01)	Patient's International Normalised Ratio. (Y-JPT)
	Lactate Dehydrogenase	-0.018 (.39)	Patient's Lactate Dehydrogenase (LD) level, measured in Units/L. (Y-JPT)
	Lymphocytes	-0.001 (.98)	Patient's Lymphocyte Count, (measured as x 10^9/L cells). (Y-JPT)
	Magnesium Level	0.024 (.27)	Patient's Magnesium level in mmol/L. (Y-JPT)
	MCV	0.073 (<.01)	Patient's Mean Cell Volume (MCV) in fL. (Y-JPT)
	Monocytes	-0.063 (<.01)	Patient's Monocyte Count, (measured as x 10^9/L cells). (Y-JPT)
	Neutrophils	-0.127 (<.01)	Patient's Neutrophil Count, (measured as x 10^9/L cells). (Y-JPT)
	Phosphate Level	0.017 (.42)	Patient's Phosphate level in mmol/L. (Y-JPT)
	Platelet Count	-0.069 (<.01)	. (Y-JPT)
	Potassium Level	-0.031 (.146)	Patient's Potassium level in mmol/L. (Y-JPT)
	Protein Total	-0.022 (.316)	Patient's total serum protein level in g/L. (Y-JPT)
	Prothrombin Time	0.042 (.055)	Patient's Prothrombin time (PT) in seconds. (Y-JPT)

	Red Cell Count	-0.054 (.013)	Patient's Red Blood Cell (RBC) Count, (measured as x 10^{12}/L cells). (Y-JPT)
	Thyroid Stimulating Hormone	0.04 (.064)	Patient's Thyroid Stimulating Hormone (TSH) level in mIU/L. (Y-JPT)
	Total HDL Chol ratio	0.01 (.655)	Ratio of Total Cholesterol to High density lipoprotein (HDL). (Y-JPT)
	Triglyceride	0.029 (.189)	Patient's Triglyceride (TG) level in mmol/L. (Y-JPT)
	Urate	0.032 (.14)	Patient's Urate level in mmol/L. (Y-JPT)
	Urea	0.014 (.521)	Patient's Urea level in mmol/L. (Y-JPT)
	Urea Creatinine	-0.02 (.354)	Urine Creatinine (mmol/24hr). (Y-JPT)
	White Cell Count	-0.117 (<.01)	Patient's White Blood Cell (WBC) Count, (measured as x 10^{9}/L cells). (Y-JPT)
Waterlow	Diabetes	0.04 (.06)	Indicates whether the patient has diabetes
	Healthy Skin	-0.072 (<.01)	Indicates whether a patient has healthy skin
	Mobility	0.028 (.2)	Patient mobility score (0-5)
	Neuro deficiency diagnosis	0.041 (.06)	Indicates whether the patient has a neurological deficiency diagnosis
	Smoking	-0.016 (.46)	Indicates whether the patient is a smoker
	WL Score	0.13 (<.01)	Transformed continuous value for patient Waterlow score. (Y-JPT)
Cluster	Features from multiple tables	-0.1 (<.01)	K-means clustering into 8 clusters (10 starting seeds) using all features except the APTT Result and cyclical datetime features.

[a]ACS: acute coronary syndrome.
[b]aPTT: activated partial thromboplastin time.
[c]ATSI: Aboriginal or Torres Strait Islander person.
[d]DVT: deep vein thrombosis.
[e]MMT: Min-Max Transformation.
[f]NZ = New Zealand.
[g]UFH: unfractionated heparin.
[h]VTE: venous thromboembolism.
[i]Y-JPT: Yeo-Johnson Power Transformation.

Table S3: Demographic data

**Feature**	**Sub-Therapeutic aPTT[a] <70 seconds**	**Therapeutic aPTT 70-100 seconds**	**Supra-Therapeutic aPTT >100 seconds**	**Total**
**Count (%)**				
All Records	1072 (49.7%)	498 (23.1%)	588 (27.2%)	2158
Gender: Male	765 (51.9%)	349 (23.7%)	359 (24.4%)	1472 (68.2%)
Gender: Female	307 (44.9%)	149 (21.7%)	229 (33.4%)	686 (31.8%)
Diagnosis[b]: Acute Coronary	337 (53.1%)	146 (23%)	152 (23.9%)	635 (29.4%)
Diagnosis**: VTE	212 (50.6%)	92 (22%)	115 (27.4%)	419 (19.4%)
**Mean (SD[c])**				
Age	64.8 (14.9)	67.6 (13.2)	67.4 (14.8)	65.8 (14.6)

Weight (kg)	90.5 (29.7)	87.1 (23.8)	83.6 (22.2)	87.8 (26.7)
Baseline aPTT (sec)	33 (10.3)	37.3 (11.1)	40.2 (11)	36 (11.1)
UFH[d] bolus dose (units)	4673 (1481)	4702 (1399)	4797 (1494)	4713 (1467)
UFH Maintenance (units)	5700 (4706)	7232 (4756)	8321 (5233)	6767 (4993)
Time between UFH bolus and aPTT (minutes)	330.5 (178.7)	394.5 (98.5)	399.8 (104.4)	364.1(149)

[a]aPTT: activated partial thromboplastin time.
[b]Diagnosis is defined using ICD-10 codes which were used to group conditions.
[c]SD=standard deviation.
[d]UFH = unfractionated heparin.

Table S4: Categorial features processing

Table	Features	Number of values	Grouping (number of groups)	Categories
Encounters' data	Medical Service patients	31	Yes (3)	Cardiology, surgery, and general medicine patients
Medications	Order Catalog	175	Yes (3)	Antimicrobial, antibiotics[b], and sedative[b] medications
Diagnosis	Diagnosis	755	Yes (3)	ACS[a], VTE[d], and Other
Powerplan	Powerplan	4	No (4)	ACS, DVT[c], Warfarin, and Low Target

[a]ACS: acute coronary syndrome.
[b]Features were discarded due to low counts.
[c]DVT: deep vein thrombosis.
[d]VTE: venous thromboembolism.

Table S5: Deleted features from pathology table due to high correlations with other features

Feature	Correlated (Deleted) Feature	Pearson Correlation
HbA1c[a] NGSP[c]	Estimated Average Glucose Level	0.996787819
HbA1c NGSP	HbA1c IFCC[b]	0.993378894
Chloride Level	Sodium Level	0.988224057
Haemoglobin	Haematocrit	0.988184823
Bilirubin conjugated	Bilirubin Total	0.959431629
Chloride Level	calcium level albumin correction	0.906861281
Chloride Level	Osmolality Calculated	0.905099402

[a]HbA1c: Hemoglobin A1C
[b]IFFC: international federation of clinical chemistry
[c]NGSP: national Glycohemoglobin standardization program
