# Supplementary material for: Predicting Therapeutic Response to Unfractionated Heparin Therapy: Machine Learning Approach
Source: Interact J Med Res. 2022 Sep 19;11(2):e34533. doi: 10.2196/34533 (PMC9531006; doi:10.2196/34533)
Supplement: Multimedia Appendix 3 [file ijmr_v11i2e34533_app3.pdf]

Multimedia Appendix 3

Part 1. Heatmaps showing correlations between different features and outcome

1- **Measurements Table:** No strong correlations were seen between height and aPTT result however a slight negative correlation occurred with weight. This slightly negative correlation of weight with aPTT at -0.095 may therefore be the result of the capping of UFH bolus dose at 2500, 5000 and 8000 units irrespective of weight.

	APTT Result	Height	Weight
APTT Result	1.000	-0.067	-0.095
Height	-0.067	1.000	0.189
Weight	-0.095	0.189	1.000

2- **Encounter Table:** The highest correlations between encounters features and aPTT result were age (plotted below) and gender, however these are still quite low at 0.13 and 0.096 respectively.

	APTT Result	Age	Aus NZ Indicator	COB Latitude	COB Longitude	Female	Hours In Hospital	Male	Married
Age	0.130	1.000	-0.127	0.204	-0.115	0.092	-0.039	-0.092	0.106
Hours In Hospital	0.000	-0.039	0.022	-0.034	0.025	0.023	1.000	-0.023	-0.063
Married	-0.004	0.106	-0.139	0.120	-0.156	-0.100	-0.063	0.100	1.000
COB Longitude	-0.003	-0.115	0.729	-0.709	1.000	0.004	0.025	-0.004	-0.156
Aus NZ Indicator	-0.018	-0.127	1.000	-0.789	0.729	0.053	0.022	-0.053	-0.139
Female	0.096	0.092	0.053	-0.046	0.004	1.000	0.023	-1.000	-0.100
Male	-0.096	-0.092	-0.053	0.046	-0.004	-1.000	-0.023	1.000	0.100
COB Latitude	-0.008	0.204	-0.789	1.000	-0.709	-0.046	-0.034	0.046	0.120

3- **aPTT Table:** Overall, the aPTT result did not correlate heavily with any feature. The strongest correlation (Pearson's coefficient of 0.291) was seen between aPTT result and the baseline aPTT. Correlations of 0.044 and 0.251 were recorded for the bolus dose and UFH maintenance, respectively.

	APTT Result	Baseline APTT	Bolus Dose	Maintenance	Minutes Betw Bolus APTT	Minutes Betw Main APTT
APTT Result	1.000	0.291	0.044	0.251	0.234	0.221
Baseline APTT	0.291	1.000	-0.152	0.211	0.158	0.182
Bolus Dose	0.044	-0.152	1.000	0.079	0.088	-0.124
Maintenance	0.251	0.211	0.079	1.000	0.309	0.878
Minutes Betw Bolus APTT	0.234	0.158	0.088	0.309	1.000	0.334
Minutes Betw Main APTT	0.221	0.182	-0.124	0.878	0.334	1.000

4- **Vital Signs Table:** No strong correlations were seen between vital sign features and aPTT, the strongest being peripheral pulse rate (-0.046). Strong correlations were seen, however, between both DBP/ SBP and AP Cuff Calc.

	APTT Result	Assisted O2	DBP	Mean AP Cuff Calc	O2 Flow Rate	Peripheral Pulse Rate	SBP	SpO2	Temperature
APTT Result	1.000	-0.029	-0.022	-0.021	-0.023	-0.046	-0.017	0.030	0.010
Assisted O2	-0.029	1.000	-0.028	-0.052	0.052	0.012	-0.068	-0.004	-0.014
DBP	-0.022	-0.028	1.000	0.822	-0.005	0.142	0.559	0.059	-0.074
Mean AP Cuff Calc	-0.021	-0.052	0.822	1.000	0.000	0.072	0.799	0.035	-0.050
O2 Flow Rate	-0.023	0.052	-0.005	0.000	1.000	0.006	-0.015	-0.074	0.113
Peripheral Pulse Rate	-0.046	0.012	0.142	0.072	0.006	1.000	-0.043	-0.067	0.141
SBP	-0.017	-0.068	0.559	0.799	-0.015	-0.043	1.000	0.051	-0.034
SpO2	0.030	-0.004	0.059	0.035	-0.074	-0.067	0.051	1.000	-0.089
Temperature	0.010	-0.014	-0.074	-0.050	0.113	0.141	-0.034	-0.089	1.000

5- **Power-plan Table:** The highest correlations between Power plan indication and aPTT result was DVT at 0.127. The ACS and low target power plans were slightly negatively correlated with aPTT value. These plans often cap the bolus dose at 5000 and 2500 units, respectively, and may be negatively correlated with APTT for this reason.

	APTT Result	ACS	DVT	Low Target	Warfarin
APTT Result	1.000	-0.088	0.127	-0.058	0.004
ACS	-0.088	1.000	-0.448	-0.245	-0.439
DVT	0.127	-0.448	1.000	-0.212	-0.380
Low Target	-0.058	-0.245	-0.212	1.000	-0.208
Warfarin	0.004	-0.439	-0.380	-0.208	1.000

6- **Diagnosis Table:** Patient diagnoses indicators again showed little correlation with aPTT result, the highest being acute coronary, which understandably has similar correlations with aPTT result as the ACS PowerPlan.

	APTT Result	Acute Coronary	Atrial Fibrillation	Mesenteric Renal	Other	PVD	Stroke	Valvular Heart	VTE
APTT Result	1.000	-0.104	-0.024	0.027	0.035	0.026	0.022	0.013	0.022
Acute Coronary	-0.104	1.000	-0.042	-0.056	-0.529	-0.150	-0.070	0.031	-0.266
Atrial Fibrillation	-0.024	-0.042	1.000	-0.019	-0.065	-0.013	0.010	-0.013	-0.044
Mesenteric Renal	0.027	-0.056	-0.019	1.000	-0.061	-0.009	0.030	-0.009	-0.053
Other	0.035	-0.529	-0.065	-0.061	1.000	-0.231	-0.081	-0.031	-0.265
PVD	0.026	-0.150	-0.013	-0.009	-0.231	1.000	0.003	-0.022	-0.033
Stroke	0.022	-0.070	0.010	0.030	-0.081	0.003	1.000	0.056	-0.022
Valvular Heart	0.013	0.031	-0.013	-0.009	-0.031	-0.022	0.056	1.000	-0.014
VTE	0.022	-0.266	-0.044	-0.053	-0.265	-0.033	-0.022	-0.014	1.000

7- **Waterlow Table:** Waterlow score showed the strongest correlation with aPTT however this was relatively low (0.118). Diabetes, healthy skin, mobility, neurological deficiency diagnosis understandably correlates strongly with WL score as they are factors that contribute to this metric.

	APTT Result	Diabetes	Healthy Skin	Mobility	Neuro Def Diag	Smoking	WL Score
Diabetes	0.025	1.000	-0.209	0.101	0.401	0.041	0.367
Healthy Skin	-0.063	-0.209	1.000	-0.259	-0.154	-0.039	-0.569
Mobility	0.030	0.101	-0.259	1.000	0.258	0.064	0.605
Neuro Def Diag	0.062	0.401	-0.154	0.258	1.000	0.057	0.478
Smoking	-0.030	0.041	-0.039	0.064	0.057	1.000	0.121
WL Score	0.118	0.367	-0.569	0.605	0.478	0.121	1.000

8- **Pathology Table:** The strongest correlations for pathology results occurred between aPTT and glucose level (-0.064), neutrophils (-0.063), INR (0.057), and bicarbonate Level (0.055) however these were rather low. White cell count had been cited in previous literature to be useful in predicting aPTT result as has INR. Very little correlation occurred between aPTT result and other pathology measures that have been previously used in studies predicting aPTT ; haemoglobin (0.011), platelets (-0.04), creatinine (-0.015), albumin level (0.025), urea (-0.04) and cholesterol (-0.04). Creatinine, haemoglobin and urea have been plotted below to highlight the box shaped dispersion of these results when compared to aPTT. Creatine is shown to be positively correlated with urea (0.684) and negatively correlated with haemoglobin (-0.048).

_	APTT Result	_	APTT Result
INR	0.057	Alanine Transaminase	0.013
Aspartate Transaminase	0.055	Lactate Dehydrogenase	0.010
Bicarbonate Level	0.055	Free T4	0.008
Bilirubin Total	0.055	Thyroid Stimulating Hormone	0.001
Bilirubin Conj	0.051	cTroponin I	0.000
Chloride Level	0.047	Alkaline Phosphatase	0.000
Sodium Level	0.046	Monocytes	-0.002
Eosinophils	0.043	Haematocrit	-0.002
Magnesium Level	0.042	Anion Gap	-0.004
Urea	0.040	Cholesterol HDL	-0.004
MCV	0.039	Haemoglobin	-0.011
Calcium Level Alb Corr	0.039	Gamma GT	-0.014
Urate	0.035	Red Cell Count	-0.014
Calcium Level	0.034	Lymphocytes	-0.014
Prothrombin Time	0.034	C Reactive Protein	-0.029
Potassium Level	0.031	HbA1c NGSP	-0.032
Urea Creat	0.030	Est Average Glucose Level	-0.035
Creatine Kinase	0.028	HbA1c IFCC	-0.036
Osmolality Calculated	0.028	Platelet Count	-0.040
Albumin Level	0.025	Cholesterol	-0.040
Phosphate Level	0.023	Triglyceride	-0.047
Protein Total	0.023	Total HDL Chol ratio	-0.048
Fibrinogen Derived	0.022	White Cell Count	-0.056
Globulin	0.017	Neutrophils	-0.063
Basophils	0.016	Glucose Level	-0.064
Creatinine	0.015		

UFH = unfractionated heparin, aPTT = activated partial thromboplastin time, VTE = venous thromboembolism. Sec = seconds, ACS = acute coronary syndrome

Part 2: Data Visualisations

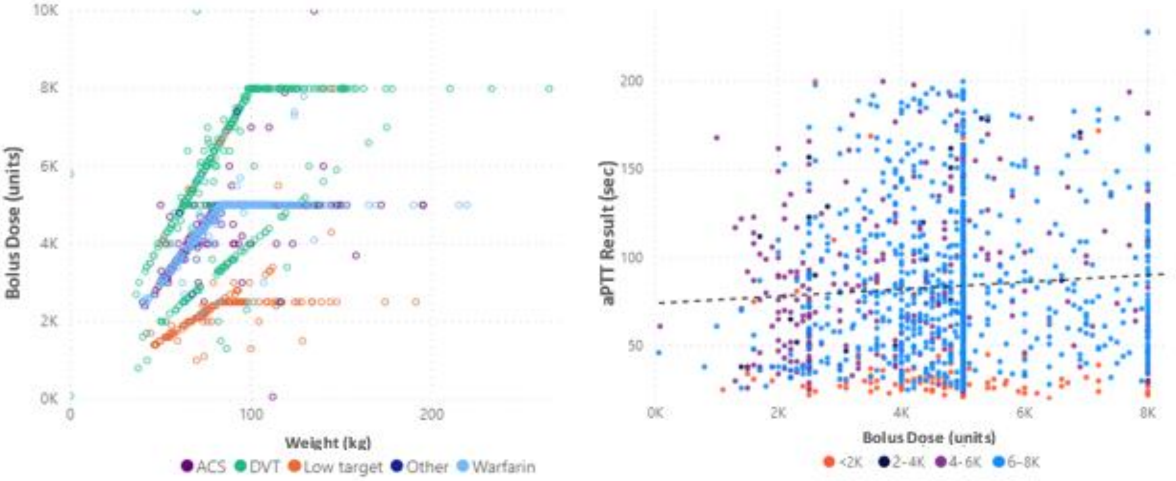

A. Weight based UFH bolus doses according to different power-plans.
B. A5b. Plot graph for UFH bolus dose and target aPTT result, colors show time in hours between the two.

UFH = unfractionated heparin, aPTT. = activated partial thromboplastin time, VTE = venous thromboembolism. Sec = seconds, ACS = acute coronary syndrome

A significant finding in this phase was the weak correlation between UFH bolus dose and the target aPTT result compared to the much stronger correlation with the UFH maintenance dose (Pearson's coefficient of 0.044 vs. 0.251). In Figure A, the UFH bolus dosing has been segmented by the type of power-plan the patient received and reveals a linear function with weight, reflecting the use of weight-based dosing by clinicians, and which varied according to the embolic indication. Consequently, the most frequent bolus dose administered was 5,000 units (ACS), 8,000 units (DVT/PE), 5000 units (warfarin replacement) and 2,500 units and for low target dosing, reflective of nomogram dose capping. This dose capping is in accordance with state-wide guidelines which were applied based on the indication for UFH. For example, for DVT/PE the maximum bolus dose is 8000 units for an 80 units/Kg bolus dose and a 1500 U/h for an 18 U/kg/h infusion. In ACS the maximum bolus is 5000 units for a 60 units /Kg bolus and 1000 U/h for a 12units/kg /hour [75]. As a result, there was very little correlation between bolus dose and target aPTT as the former was constant for many patients treated using a power plan.

Figure B shows a scatter plot reflecting the relationship between UFH bolus dose and the corresponding target aPTT result according to time interval between the two (grouped by 2-hour intervals). The majority of aPTT tests were done between 4 and 8 hours after the bolus dose, and many of the aPTT results below 50 seconds occurred when the bolus dose was administered within 2 hours of the test (orange), at which time the full effect of the dose has not been realised. The time between bolus dose and aPTT contributed to a modelling feature (UFH bolus time calculated) whereby the bolus dose was divided by the minutes between the bolus dose and subsequent aPTT.

Part 3: Predictions' visualisation using residual graph

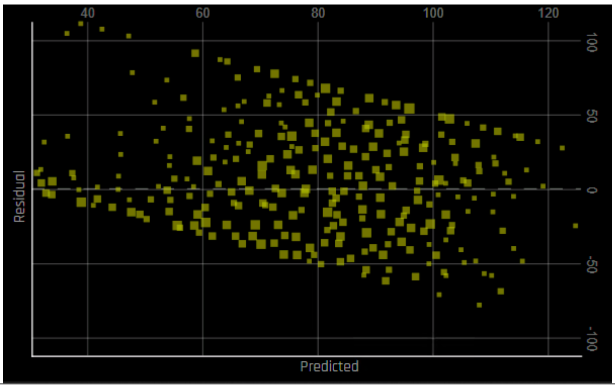

Figure 3A: target floor & ceiling applied

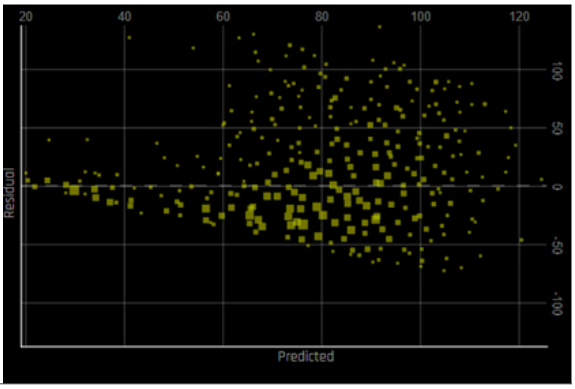

Figure 3B: no target floor & ceiling applied

This figure shows most of the highest residuals occurring at the high and low end of the predictions. Looking at the non-capped version of this visual can be seen several residuals over 100 or near it (most notably on high aPTT predicted values) but compared to the capped model there is hardly any residuals near this.
