# Supplementary material for: Predicting Therapeutic Response to Unfractionated Heparin Therapy: Machine Learning Approach
Source: Interact J Med Res. 2022 Sep 19;11(2):e34533. doi: 10.2196/34533 (PMC9531006; doi:10.2196/34533)
Supplement: Multimedia Appendix 4 [file ijmr_v11i2e34533_app4.pdf]

Multimedia Appendix 4

$$RMSE = \sqrt{\frac{\sum_{i=1}^{N}(\hat{y_i} - y_i)^2}{N}}$$

$$MAE = \frac{\sum_{i=1}^{N}(\hat{y_i} - y_i)}{N}$$

$$R^2 = 1 - \frac{\sum_{i=1}^{N}(y_i - \hat{y_i})^2}{\sum_{i=1}^{N}(y_i - \bar{y})^2}$$

where N is the number of records, y is the outcome column, $\bar{y}$ in mean value of y, and $\hat{y}$ is the predicted column of y.

$$Accuracy = \frac{\sum_{k=1}^{K} M_{k,k}}{\sum_{i=1}^{K}\sum_{k=1}^{K} M_{i,k}}$$

$$Macro\ Precision = \frac{\sum_{k=1}^{K} Precision_k}{K}, Precision_k = \frac{TP_k}{TP_k + FP_k}$$

$$Macro\ Recall = \frac{\sum_{k=1}^{K} Recall_k}{K}, Recall_k = \frac{TP_k}{TP_k + FN_k}$$

$$Macro\ F1\ Score = \frac{2 \times Macro\ Precision \times Macro\ Recall}{Macro\ Precision^{-1} + Macro\ Recall^{-1}}$$

$$Macro\ AUC = \frac{\sum_{k=1}^{K} AUC_{k,rest_k}}{K}$$

where K is the number of classes, and M is the confusion matrix.

The Yeo-Johnson power transformation [74] was defined by:

$$\psi(\lambda, y) = \begin{cases} \left((y+1)^{\lambda} - 1\right)/\lambda & ,\lambda \neq 0, y \geq 0 \\ \log(y+1) & ,\lambda = 0, y \geq 0 \\ -\left((-y+1)^{2-\lambda} - 1\right)/(2-\lambda) & ,\lambda \neq 2, y < 0 \\ -\log(-y+1) & ,\lambda = 2, y < 0 \end{cases}$$

where y is a list of numbers, and λ (lambda) is the transformer whose value is determined by the model to maximise the log-likelihood function.

The equation to calculate external validation sample is as follows:

$$n = Max\left(\frac{4R^2\ (1-R^2)^2}{SE^2_{R^2}}, \frac{var(Y_i)(1-R^2)}{SE^2_{CITL}}, \frac{\lambda^2_{cal}(1-R^2)}{SE^2_{\lambda_{cal}}R^2}+1, MMOE = \sqrt{Max\left(\frac{\chi^2_{1-\frac{a}{2},n-1}}{n-1}, \frac{n-1}{\chi^2_{\frac{a}{2},n-1}}\right)}\right)$$

$$R^2 = 1 - \left(\frac{var(Y_i - Y_{PREDi})}{var(Y_i)}\right)$$

where R^2 is the proportion of outcome variation calculated from the proposed model in our external validation experiment ($R^2 = 0.365$), SE stands for the standard error, for which we used the value 0.051 for $SE_R{}^2$ to achieve a CI of 90% [57]. The variance in our EV dataset ($var(Y_i) = 1472$), and we estimated an acceptable value for ($SE_{CITL} = 4.16$). CITL stands for calibration-in-the-large, to keep the prediction spread in the range of ±12.5. For the third part of the equation, we choose a calibration value ($\lambda_{cal} = 1$) as recommended by the authors, and ($SE_{\lambda cal} = 0.1$) to achieve a CI of 90%. The last part of the equation is multiplicative margin of error (MMOE) within 10% margin of error, as recommended by the authors. The equation generated a sample size of at least 235 participants (Table 4) which was achieved.

Table 4. Calculating minimum size of external validation dataset

$\frac{4R^2\ (1-R^2)^2}{SE^2_{R^2}}$	$\frac{var(Y_i)(1-R^2)}{SE^2_{CITL}}$	$\frac{\lambda^2_{cal}(1-R^2)}{SE^2_{\lambda_{cal}}R^2}+1$	$MMOE$	n
226	225	175	235	235

MMOE = multiplicative margin of error, Var = variance, n = number, SE = standard error.
